# Influence of working in auto factory on gastroesophageal reflux disease 

**Published:** 2018

**Authors:** Hadis Najafimehr, Sara Ashtari, Hamid Mohaghegh Shalmani, Zeinab Fazeli, Hosein Yadegari, Hamed Taherinejad, Khosrow Manhoie, Seyed Ramin Rasooli, Abbas Moradi, Mohamad Javad Akbariju, Hosein Mohseni, Maryam Nasserinejad

**Affiliations:** 1 *Basic and Molecular Epidemiology of Gastrointestinal Disorders Research Center, Research Institute for Gastroenterology and Liver Diseases, Shahid Beheshti University of Medical Sciences, Tehran, Iran*; 2 *Gastroenterology and Liver Diseases Research Center, Research Institute for Gastroenterology and Liver Diseases, Shahid Beheshti University of Medical Sciences, Tehran, Iran*; 3 *Department of Occupational Health & Safety, Company of SAIPA Automotive Corporation, Tehran, Iran*; 4 *Department of Administrative Management, Company of SAIPA Automotive Corporation, Tehran, Iran *

**Keywords:** Gastroesophageal reflux disease, Risk factor, Work shift, Gastrointestinal cancer, Peptic ulcer

## Abstract

**Aim:**

Present study aimed to evaluate association between job -related factors and gastroesophageal reflux disease (GERD) among Iranian auto factory’s workers.

**Background:**

Many of the gastrointestinal disorders may be caused as the result of stress-related occupations and biorhythm disruption.

**Methods:**

We performed a cross-sectional study on 3590 Iranian Auto factory employees. GERD symptoms, demographic information, work shift, work section and history of some gastrointestinal disease were asked from all employees by physician. Logistic regression was used to estimate the odds ratios (OR) and 95% confidence intervals (CI) for GERD symptoms according to the potential risk factors.

**Results:**

The prevalence of GERD was 25.57%, which was higher in rotatory shift (91.6%) than the fixed shift (8.4%) (P-value = 0.009). Smoking (OR: 1.31; 95% CI: (1.09, 1.57)), working in official section (P-value < 0.001), history of GERD (OR: 8.63; 95 % CI (6.53, 11.40)), history of peptic ulcer (OR: 2.96; 95 % CI (2.08, 4.20)), family history of gastrointestinal cancers (OR: 1.47; 95 % CI (1.19, 1.81)) were the factors associated with GERD symptoms.

**Conclusion:**

The prevalence of GERD in the rotatory shift was more than the fixed shift. Smoking, family history of gastrointestinal cancers and peptic ulcer could be associated with GERD symptoms. Working in the special job with high activity, may probably lead to decrease in the risk of reflux.

## Introduction

 Gastroesophageal reflux disease (GERD) is one of the gastrointestinal disorders which is occurred when esophagus meets gastric acid (1) and results from abnormal movement of stomach contents upward (2). GERD is one of the most prevalent diseases in the world and its prevalence does not have a homogeneous distribution worldwide. The researches have reported that the GERD prevalence in the North American is up to 28%, in the Europe up to 26%, in the Asia up to 33% and in the Middle East up to 12% (3-5). Previous study revealed that in Iran, the GERD prevalence is increasing by the time and admission of GERD patients has been increased 3 folds at the short time (6). The GERD treatment has strained heavy costs to governments. At 2000, this costs in the United State was near to 8 billion dollars and in the United Kingdom was 461 million pounds (7, 8). In Iran the total direct costs of reflux disease per patient is estimated as PPP$97.70 (PPP, purchasing power parity dollars) and the total indirect cost of disease per patient is PPP$13.7 (9). The early treatment of GERD is very important because of producing painful complications and, it can lead to serious consequences in the future. Disruptive GERD has a high burden of disease compared with occasional or mild reflux symptoms (10). The precipitating factors of GERD are obesity, smoking, alcohol consuming and non-steroidal anti-inflammatory drugs (NAIDS) usage. Additionally, some foods such as hot drinks, pepper food, citrus, onion, tomato and pepper mint and also some habits such as immediately sleeping after food consuming, may intensify the GERD symptoms (11-13). Furthermore, Stress through an impact on the immune and nervous body system can be important factor. Previous studies have shown that occupational stress has a direct impact on GERD symptoms (14). 

The concept of work shift, actually was introduced after industrial revolution and nowadays many people are working at day or night shift (15). But it is obvious that working at night, by disruption in circadian rhythm, may have negative effect on health and gastrointestinal regulatory processes (16). Many of the gastrointestinal disorders may be caused as the result of by stress-related occupations and biorhythm disruption (14, 17). Gastrointestinal disorders such as GERD can cause loss of work, and besides treatment costs, impose indirect costs on employers (18). 

There is a few studies on verifying the relation between shift working and GERD (19) while, the work- related factors, may be essential in GERD prevention. This study was designed to evaluate the prevalence of GERD and its precipitating factors, especially job- related factors, in a large auto factory, as a first study in Iran. 

## Methods

**Participant and questionnaire**

This study is a part of gastrointestinal case finding survey and was done on workers in Saipa Auto factory, located in Tehran suburb, in 2017. In this cross-sectional survey, all workers invited to participate in the study; then workers who had consent, collaborated with the study so, the sample size was 3590 and the method of sampling was convenience sampling (20). Most of workers are male, so only male workers enrolled in the study. Workers of different sections of factory, including Administrative and Support (A), Paint (P), Montag (M), Body (B) and Press & Platform (P&P), were evaluated. According to shift schedule, they were divided into two categories: fixed shift (7:00 am to 15:30 pm) and rotating shift. Rotating shift is a scheduling system where workers each week move through a cycle of working on day shift (7:00 am to 15:30 pm), evening shift (16:00 pm to 24:00 am), and night shift (24:00 am to 07:00am). Workers usually worked 7 days per week; Saturday to Thursday. Working shift in the last 3- month was considered as workers’ shift time. 

A questionnaire, including demographic data, habits, gastrointestinal manifestations within last 3- month, past medical history, family history, and drug history was fulfilled for each worker via face to face interview. Gastrointestinal (GI) symptoms, including abdominal pain/discomfort, constipation, acute diarrhea, chronic diarrhea, bloating/ flatulence, heartburn/acid regurgitation, decreased appetite, nausea/vomiting, bloody or black stool (melena), anorexia/weight loss, and difficulty of swallowing were asked. The body mass index (BMI) was categorized according to definition by world health organization (WHO) (BMI of less than 18.5 is considered as underweight, BMI equal to or greater than 25 is considered overweight and above 30 is considered obese) (21). 

**Table1:** Characteristics of 3590 Auto factory workers according to demographic, work features and disease history (in general and in the work shifts)

	Total	Fixed shift N= 379	Rotating shift N=3170	P- value++
Variables				
Age				0.99
20-29	252 (7.3)	27 (7.4)	225 (7.3)	
30-39	2448 (70.6)	258 (70.7)	2190 (70.6)	
40-49	728(21.0)	76 (20.8)	652 (21.0)	
≥50	37(1.1)	4 (1.1)	33 (1.1)	
BMI*				
<25	1374 (40.0)	123 (34.5)	1251 (40.6)	0.02+
25-29.9	1784 (51.9)	195 (54.6)	1589 (51.6)	
≥30	278 (8.1)	39 (10.9)	239 (7.8)	
Smoking				0.31
Yes	878 (24.7)	102 (26.9)	776 (24.5)	
No	2671 (75.3)	277 (73.1)	2394 (75.5)	
Exposure to chemical				< 0.001+
Yes	983 (27.7)	16 (4.2)	967 (30.5)	
No	2566 (72.3)	363 (95.8)	2203 (69.5)	
Work section				< 0.001+
Administrative & supporting	31 (0.9)	22 (5.8)	9 (0.3)	
Paint	816 (23.0)	7 (1.8)	809 (25.5)	
Montage	1468 (40.8)	51 (13.5)	1397 (44.1)	
Body	979 (27.6)	241 (63.6)	738 (23.3)	
Press & platform	275 (7.7)	58 (15.3)	217 (6.8)	
History of GERD				< 0.001+
Yes	315 (8.8)	17 (4.5)	298 (9.4)	
No	3234 (91.1)	362 (95.5)	2872 (90.6)	
History of PU**				0.16
Yes	167 (4.7)	12 (3.2)	155 (4.9)	
No	3382 (95.3)	367 (96.8)	3015 (95.1)	
History of GC *+				1.00
Yes	46 (1.3)	5 (1.3)	41 (1.3)	
No	3503 (98.7)	374 (98.7)	3129 (98.7)	
Family history of GC				0.37
Yes	563 (15.8)	66 (17.4)	497 (15.7)	
No	2986 (84.2)	313 (82.6)	2673 (84.3)	

* : Body mass index;

** : Peptic ulcer;

*+ : Gasrtointestinal cancers;

++ : Using Chi- square test;

+ : Significant.

All questionnaires were evaluated by a physician. Those who need further evaluation were asked for laboratory tests and or imaging, and if need, referred to gastroenterologist. Finally, GI symptoms, and GI diagnoses were dependent variables.

In our study, GERD was defined as heartburn and/or acid regurgitation occurring at least weekly (22).

**Statistical analysis**

We compared the possible risk factors for GERD and the demographic information of workers according to the shift which they were working. Also the prevalence of GERD between different work shifts was compared by using the chi-squared test. The association between each variables and GERD was analyzed with the aid of univariate logistic regression model and the obtained significant variables were imported to the multivariate model. The odds ratios (ORs) and confidence intervals (CIs) were reported for both models. All statistical analyses were performed by using the SPSS 21 software. The significant level was considered as 0.05.

**Table 2:** Factor associated with Gastroesophageal reflux disease (GERD) among 3590 Auto factory workers

Variable	Normal (n= 2672)	GERD (n= 918)	Univariate		multivariate££	
			OR£ (95% CI)	P- value++	OR (95% CI)	P- value++
Age				0.27		
< 40	2015 (77.5)	714 (79.2)	1			
≥ 40	586 (22.5)	187 (20.8)	0.90 (0.75, 1.08)			
BMI*				0.83		
< 25	1038 (39.9)	352 (39.5)	1			
≥ 25	1566 (60.1)	540 (60.5)	1.02 (0.87, 1.19)			
Smoking				0.002+		0.005+
Yes	623 (23.3)	261 (28.4)	1.31 (1.10, 1.55)		1.31 (1.09, 1.57)	
No	2049 (76.7)	657 (71.6)	1		1	
Exposure to chemical				0.02+		0.61
Yes	710 (26.6)	280 (30.5)	1.21 (1.03, 1.43)		0.92 (0.67, 1.27)	
No	1962 (73.4)	638 (69.5)	1		1	
Work shift				0.009+		0.30
Fixed shift	303 (11.5)	76 (8.4)	1		1	
Rotating shift	2338 (88.5)	832 (91.6)	1.42 (1.09, 1.85)		1.17 (0.86, 1.59)	
Work section				< 0.001+		0.001+
Administrative & supporting	16 (0.6)	15 (1.6)	1		1	
Paint	591 (22.1)	229 (24.9)	0.41 (0.20, 0.85)	0.02+	0.42 (0.18, 0.97)	0.04+
Montage	1015 (38.0)	451 (49.1)	0.47 (0.23, 0.97)	0.04+	0.41 (0.19, 0.92)	0.03+
Body	817 (30.6)	175 (19.1)	0.23 (0.11, 0.47)	< 0.001+	0.29 (0.13, 0.65)	0.003+
Press & platform	233 (8.7)	48 (5.2)	0.22 (0.10, 0.47)	< 0.001+	0.27 (0.18, 0.63)	0.002+
History of GERD				< 0.001+		< 0.001+
Yes	81 (3.0)	235 (25.6)	11.00 (8.43, 14.36)		8.63 (6.53, 11.40)	
No	2591 (97.0)	683 (74.4)	1		1	
History of PU**				< 0.001+		< 0.001+
Yes	73 (2.7)	95 (10.3)	4.11 (3.00, 5.63)		2.96 (2.08, 4.20)	
No	2599 (97.3)	823 (89.7)	1		1	
History of GC *+				< 0.001+		0.16
Yes	18 (0.7)	28 (3.1)	4.64 (2.55, 8.42)		1.65 (0.82, 3.35)	
No	2654 (99.3)	890 (96.9)	1		1	
Family history of GC				< 0.001+		< 0.001+
Yes	380 (14.2)	188 (20.5)	1.55 (1.28, 1.88)		1.47 (1.19, 1.81)	
No	2292 (85.8)	730 (79.5)	1		1	

* : Body mass index;

** : Peptic ulcer;

*+ : Gasrtointestinal cancers;

£ : Odds ratio;

++ : Using logistic regression;

+ : Significant;

££ : Adjusted model

## Results

A total of 3590 male workers with mean (± standard deviation) age of 36.46 ± 4.09 years and mean BMI of 25.90 ± 2.81 Kg/m^2 ^were enrolled in the study. Among the workers there were no body with underweight condition. The descriptive statistics for all workers according to work shifts are shown in table 1. The number of workers on fixed shift and rotating shift was 379 (10.7 %) and 3170 (89.3 %), respectively. The prevalence of BMI > 25 in the rotating shift (in the 59.4 % of workers) was significantly lower than the fixed shift (in the 65.5 % of workers) (*P*= 0.02). The exposure to chemical materials in the rotating shift was more than the other shift (*P*< 0.001). The workers in different parts of the work did not have a uniform distribution between two shifts (*P*<0.001). The previous history of GERD between two types of shifts was different (*P*<0.001). Peptic ulcer, history of gastrointestinal cancers and family history of gastrointestinal cancers did not have any significant association with work shift (*P*>0.05). Forty six workers had different gastrointestinal cancers; 5 esophageal, 9 colorectal, 6 liver, and 26 gastric cancer.

The total prevalence of GERD was 25.57 % (95 % CI: 22.80, 28.40) among all workers. While, this prevalence for workers with rotating shift and fixed shifts was 26.2% and 20.1%, respectively. The majority of workers with GERD was workers with rotating shift (91.6%), the association between GERD and work shift was significant (*P*= 0.009).

Table 2 represents the OR and 95 % confidence interval (CI) of GERD association with its risk factors such as work shift. In univariate logistic regression, work shift was significantly associated with GERD (OR: 1.42; 95 % CI: (1.09, 1.85)) and the chance of GERD in Administrative & supporting (Official) section was more than the others (Non-official) (*P*< 0.001). Smoking (OR:1.31; 95 % CI: (1.10, 1.55)), Exposure to chemical materials (OR: 1.21; 95 % CI: (1.03, 1.43)), History of GERD (OR: 11.00; 95 % CI: (8.43, 14.36)), History of peptic ulcer (PU) (OR: 4.11; 95 % CI: (3.00, 5.63)), history of Gastrointestinal cancers (GC) (OR: 4.64; 95 % CI: (2.55, 8.42)), family history of GC (OR: 1.55; 95 % CI: (1.28, 1.88)) were significantly associated with GERD. Multivariate logistic regression model controlling for significant mentioned covariates, revealed that smoking (OR: 1.31; 95 % CI: (1.09, 1.57)), history of GERD (OR: 8.63; 95 % CI: (6.53, 11.40)), history of PU (OR: 2.96; 95 % CI: (2.08, 4.20)), family history of GC (OR: 1.47; 95 % CI: (1.19, 1.81)) and also work section (*P- value* < 0.001) were significantly associated with GERD.

## Discussion

Since lifestyle and environmental conditions play important roles in the development of disease, the association between occupational status and community health in the management of health services is essential (23). There are several studies that have identified the precipitating factors of GERD (24) however, a few studies have been conducted on job-related risk factors (25). The aim of the present study was to evaluate the association between GERD, different part of work sections and shift schedules. In addition, we evaluated the impact of some disease history on GERD among Iranian Auto factory’s workers. 

Heart burn (sensation of burning in retrosternal part of body) and acid regurgitation (an acidic tasting in the mouth) are principal GERD symptoms that probably many people have experienced during their life; however, this feeling is considered as a complication if it is frequent and severe (26, 27). It should be noted that in the present study, all workers were evaluated by the physician for diagnosis of gastrointestinal disorders and reflux symptoms, and then the authors used confirmed data instead of the self-report data.

Global comparison of prevalence, shows the variety rate of reflux prevalence. The prevalence in the Western countries is more than Europe and Asia (26, 28). In Iran, as an Asian country, there are several studies that investigated GERD prevalence in the different areas after 2005 (29). In an Iranian study by Nouraie *et al.* the prevalence of GERD was reported 18.4% (30). In another study by Firoozi *et al.* the GERD prevalence in Shiraz (South of Iran) was reported 15.4% (31). In the present study, the prevalence of GERD among the workers was 25.57% which is more than the previous studies. It is important to note that previous studies were conducted on healthy random population, whereas, our study was on employees. It is clear that there are some differences between random population and workers with special condition in aspect of physical activity and stress situation (32). 

In the current study, the association between BMI and shift schedules was significant and BMI for workers with rotating shift (mean: 25.85 Kg/m^2^) was less than the workers with fixed shift (mean: 26.25 Kg/m^2^). Similarly, in a study by Fesharaki *et al*. that investigated the night shift workers, a negative association was confirmed (33). In another cross-sectional research by Chung *et al. *on 6040 Korean shipyard workers which was designed to evaluate the association between night shift work and erosive reflux disease symptoms, the BMI association was not significant (34). In our study, the smoking did not have any significant association with the shift work, as similar as the results of Chung *et al*. study (34). 

According to our result, the univariate logistic regression showed a positive significant association between rotating shift and GERD symptoms (OR: 1.42; 95 % CI (1.09, 1.85). while the result of multivariate logistic regression in Chung *et al.* (34) study for this association (OR: 1.41; (1.03, 1.94)), is very close to our univariate result, in the present study multivariate model has not confirmed any significant association (OR: 1.17; 95 % CI (0.86, 1.59)) for shift schedules. Its reason may be due to some differences between the nature of night shift in Chung *et al.* (34) research and rotating shift in our study. The workers in our study were worked in the rotating situation at day and sometimes at night. The second reason is different variables which were included in our multivariate model and affected the associations. Addition to the shift schedules, we evaluated the impact of work in different parts of factory on GERD. Our result elucidated that the association between GERD and work section was statistically significant (*P*<0.001) and the chance of GERD for workers who were working in Non-administrative section, was less than the other sections (OR<1). In the present study the multivariate logistic regression revealed a positive association between GERD symptoms and smoking (OR of GERD in smokers was higher than the non- smokers by 31%). The Chung *et al.* (34) study also has reported the similar association. The results of our study also revealed that previous history of GERD (OR: 8.63; 95% CI (6.53, 11.40)), history of peptic ulcer (OR: 2.96; 95 % CI (2.08, 4.20)) and also family history of gastrointestinal cancers (OR: 1.47; 95 % CI (1.19, 1.81)), are the factors associated with GERD symptoms. About reflux history, it has been proven, in patients with unrecognized disease or those who have not completed the duration of treatment, GERD may lead to a chronic disease and also may cause some complications (11). Our results confirmed the importance of timely treatment. The previous studies on gastrointestinal ulcer, have reported that some gastrointestinal disorder due to gastric acid, is related to duodenal ulcer (35) and this point is confirmed by our retrospective research; however, it is possible that reflux had led to peptic ulcer in the workers. About gastrointestinal cancers, previous researches on adenocarcinoma of the esophagus have showed an increasing in the cancers incidence from Barrett's esophagus in some parts of the world and those proved that there is an association between Barrett’s reflux and adenocarcinoma (36). The result of a meta-analysis for patients with weekly symptoms of GERD revealed that the odds of esophageal adenocarcinoma significantly increases up to five fold (37). In the present study, all workers were evaluated for all gastrointestinal cancers including esophageal cancer but the association between GERD and history of caners was not significant. Perhaps the low cases of cancerous patients is the reason of this finding. 

In our study the psychological factors such as stress was not investigated and this point may be considered as a limitation. In addition, our results for history of disease were based on self- report and the result may be affected by the recall bias. On the other hand, to the best of our knowledge, this is the first study in Iran that evaluated the relationship between occupational factors and GERD symptoms. Future studies to investigation of job factors with controlling for food habitation is suggested (38). 

In conclusion, the present study revealed that addition to job- related variables, previous history of gastrointestinal cancers and peptic ulcer, may be the factor associated with GERD. Working in the special job with high activity probably decreases the risk of reflux.
